# Initial experience with triple port laparoscopic distal gastrectomy

**DOI:** 10.3389/fonc.2022.1042314

**Published:** 2023-01-27

**Authors:** Jiangpeng Wei, Xisheng Yang, Ruiqi Gao, Weidong Wang, Xiaohua Li, Gang Ji

**Affiliations:** Department of Gastrointestinal Surgery, Xijing Hospital of Digestive Diseases, Fourth Military Medical University, Xi’an, China

**Keywords:** gastric cancer, TPLDG, FPLDG, distal gastrectomy, laparoscopic surgery

## Abstract

**Objective:**

This study aimed to compare the feasibility and short-term clinical efficacy of triple-port laparoscopic distal gastrectomy (TPLDG) with five-port laparoscopic distal gastrectomy (FPLDG).

**Methods:**

From April 2020 to December 2021, this retrospective study included all consecutive patients (n = 21) who underwent TPLDG + D2 lymph node dissection, and randomly screened patients who underwent FPLDG + D2 lymph node dissection during this period (n = 30).

**Results:**

There were no significant differences in intraoperative (*P* > 0.05) and postoperative complication rate (*P* = 0.635) between the two groups. The changes in the first ambulation, flatus, water intake after surgery and postoperative hospitalization were also similar between the two groups (*P* > 0.05). However, time to abdominal drainage tube removal (1.62 ± 0.15 days vs. 2.00 ± 0.12 days, *P* = 0.046), NRS pain score on the first postoperative day (1.91 ± 0.15 days vs. 2.47 ± 0.12 days, *P* = 0.004) and hemameba level on the third postoperative day (7.89 ± 0.51 days vs. 10.52 ± 0.58 days, *P* = 0.002) were significantly lower in the TPLDG group compared to the FPLDG group.

**Conclusion:**

TPLDG is a safer, feasible, and short-term alternative to conventional LDG for distal gastric cancer.

Gastric cancer (GC) is the one of most common cancers and a leading cause of cancer-related deaths worldwide (1). During past decades, the mortality of gastric cancer had decreased significantly in most places due to the development of modern medicine. Gastrectomy with lymph node (LN) dissection is the main surgical treatment area for non-metastatic gastric cancer (2, 3). Among several treatment options, laparoscopic gastrectomy has been widely performed and has been reported by various studies regarding its advantages, such as better postoperative outcomes to open gastrectomy (4). In order to promote the development of laparoscopic methods, experienced surgeons have now begun to investigate the use of reduced-port laparoscopic surgery for gastric cancer, which not only enhances patient recovery after surgery but also results in a decrease in both pain and cost (5). Several recent studies have reported favorable surgical outcomes for reduced-port laparoscopic gastrectomy (6–8).

One of the reduced port surgery options, single-incision laparoscopic surgery plus one port (SILS+1), in which fewer ports and smaller incisions are needed, has become increasingly popular in the past few years. However, several problems, such as the “coaxial effect”, precise teamwork cooperation and other technical difficulties of operation, significantly limit the popularity of SILS+1 (7, 9, 10). Based on the idea of SILS+1 and improving its shortcomings, our team proposed a new technique named triple-port laparoscopic distal gastrectomy (TPLDG) for early gastric cancer or partial advanced lower gastric cancer. To the best of our knowledge, there are no previous reports on the use of TPLDG for total laparoscopic distal gastrectomy. Therefore, this report aims to compare the feasibility and short-term clinical efficacy of TPLDG with five-port laparoscopic distal gastrectomy (FPLDG).

## 1 Material and methods

### 1.1 Patients

This study recruited patients who were treated with TPLDG + D2 lymph node dissection from April 2020 to December 2021 consecutively, and randomly screened patients who underwent FPLDG + D2 lymph node dissection during this period (n = 30) in the department of gastrointestinal surgery of the First Affiliated Hospital of Air Force Military Medical University.

All characteristics of patients were obtained from the database in a retrospective view, which included demographic features, pathologic results, operative data, and postoperative outcomes. Demographic features included age (18-70 years), body mass index (BMI) < 24 kg/m^2^, pathology-confirmed gastric adenocarcinoma, and complete clinical data of the patient. Operative data involved tumor finding or location (middle and/or lower stomach), tumor diameter ≤ 3 cm, preoperative clinical stage cT_1b_ ~ T_2_N_0_ ~ N_1_M_0_ according to the 7th edition of the AJCC Cancer Staging Manual (gastric cancer) (11), Eastern Cooperative Oncology Group score 0 to 1 (11), procedures of distal radical gastrectomy, and American Society of Anesthesiologists class I to II. Postoperative outcomes referred to perioperative management according to the accelerated rehabilitation surgical process. The following criteria were not included: (1) history of previous upper abdominal surgery (except cholecystectomy); (2) presence of other malignancies; (3) cases converted to laparotomy. Importantly, the patients and family members gave written informed consent for surgery prior to the operation.

### 1.2 Surgical approach

**Figure 1:**
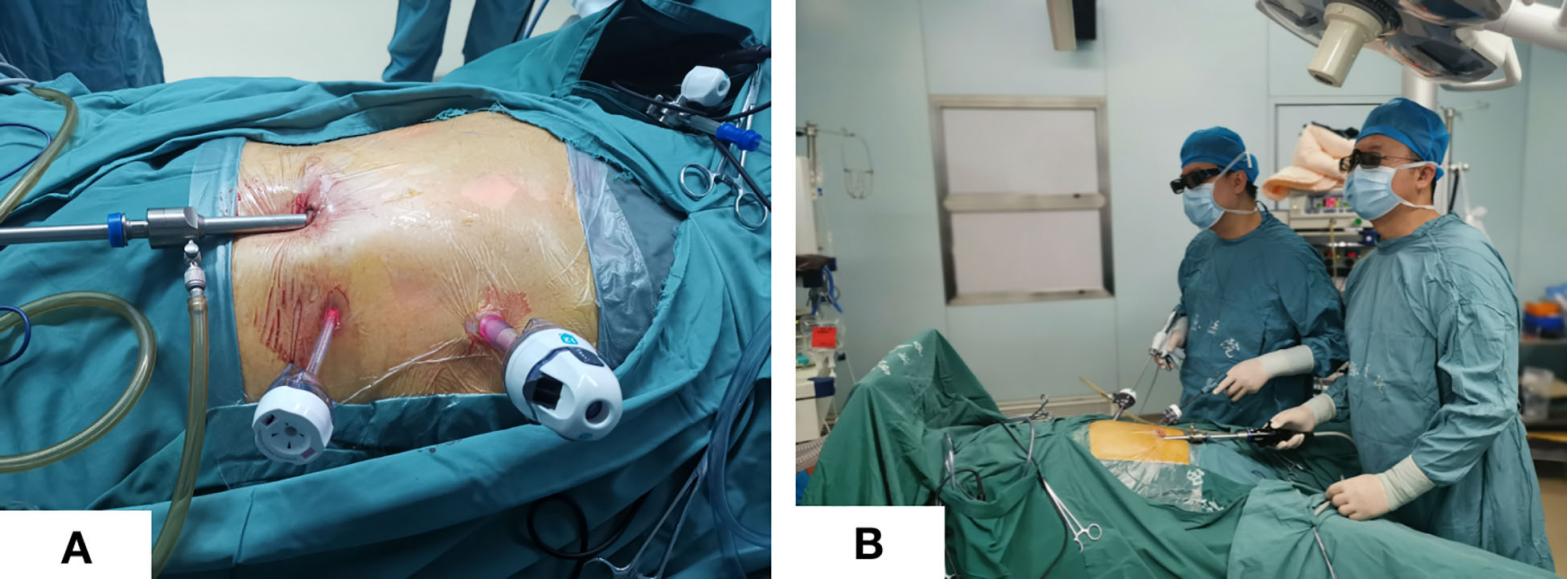
Trocar placement and surgeon position. **(A)** Trocar placement; **(B)** Standing position of the surgeon and assistant.

The patient was placed in the lithotomy position with legs apart. A 12 mm trocar was placed in an incision 2 cm below the costal margin of the left anterior axillary line (**Figure 1A**). The surgeon stood on the patient’s left side (**Figure 1B**), and the assistant held the mirror stands between the patient’s legs. An upward and arc-shaped 1 cm incision was made below the umbilicus, followed by a 0.5cm incision on the midclavicular line just outside of the umbilicus, in which a 5 mm trocar was placed.

Surgical quality control and main operating points were referred to Surgeon Quality Control and Standardization of D2 Lymphadenectomy for Gastric Cancer (10). Almost all procedures were performed in the same manner as those in conventional laparoscopic gastrectomy (CLG). The uses of the forceps in the operator’s left and right hand were the same as CLG, and liver retraction was performed routinely.

**Figure 2:**
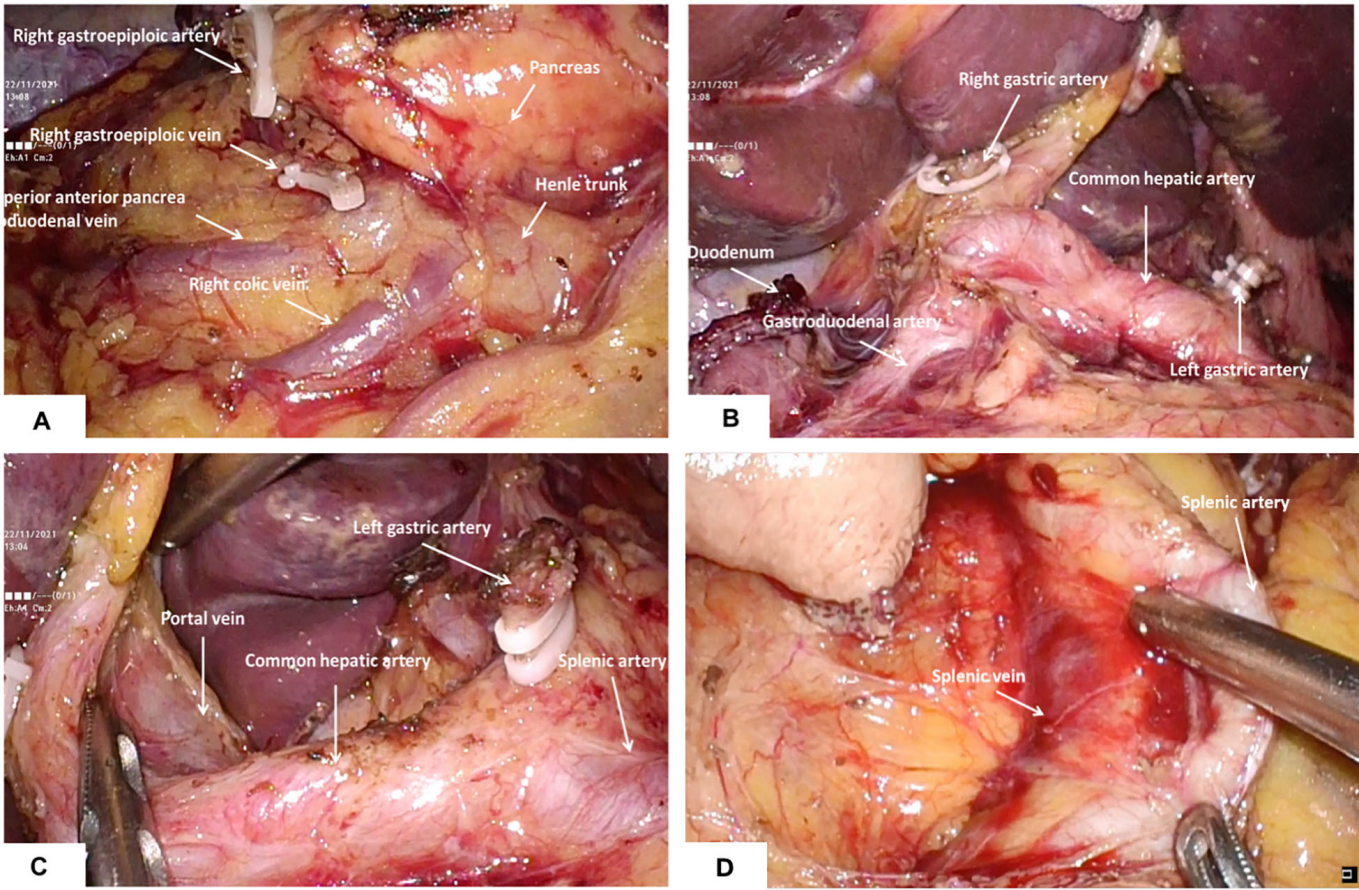
Dissection of perigastric lymph nodes **(A)** the right gastroepiploic artery and vein were identified and divided at the base to dissect the infrapyloric lymph nodes; **(B)** The lymph nodes around the right gastric artery were dissected; **(C)** Exposure of portal vein and dissection of lymph nodes in No.12; **(D)** The splenic artery was exposed and the No. 11p lymph nodes were cleaned.

**Figure 3:**
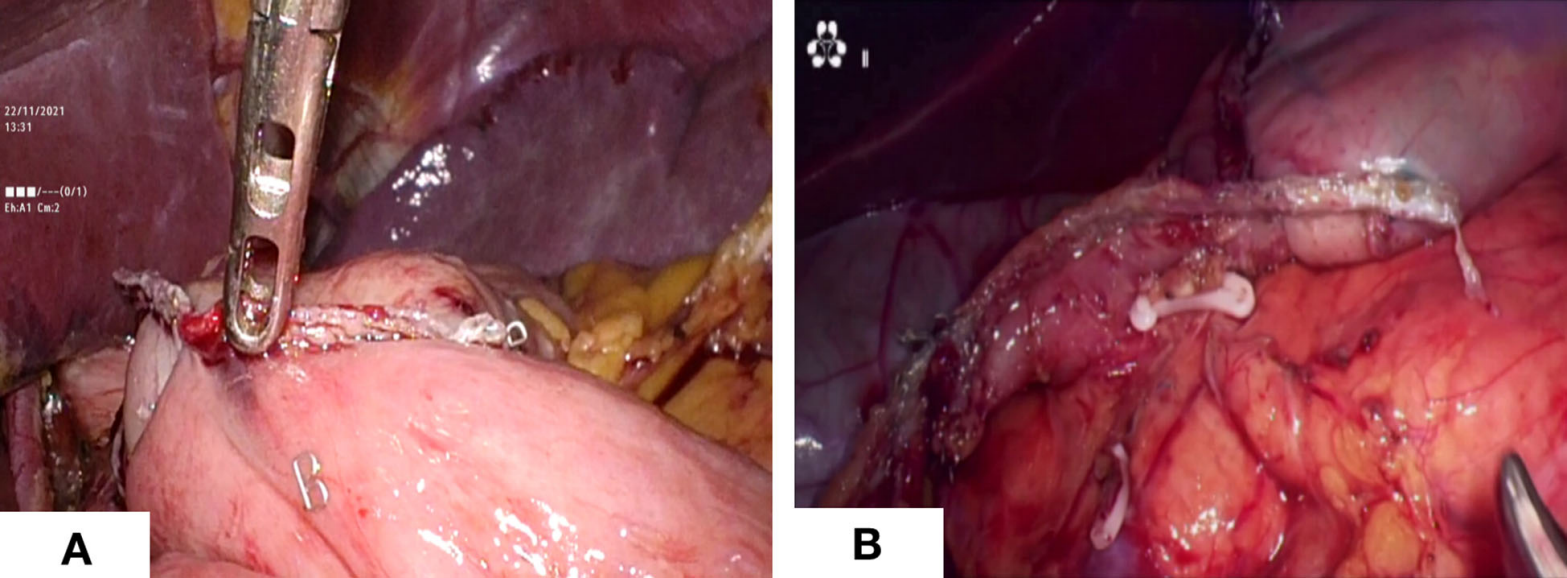
Digestive tract reconstruction. **(A)** Billroth -I anastomosis; **(B)** Billroth - II anastomosis.

The sequences of perigastric lymph node dissection vary, and some unique procedures need to give priority. Firstly, the omentum was lifted using duckbill forceps in the left hand and the mesocolon was dissected using a harmonic in the right hand. It is significant for the surgeon to create the working field by fully using the pulling effect of both hands. The anatomy-based approach made lymph node dissection easier. Then, the right side omentum and gastric tissue were transferred to the left to fully expose the gastroomental arteriovenous area (**Figure 2A**). In the meanwhile, the non-working surface of the ultrasonic knife was close to the splenic artery and tentatively picked up the lymphatic tissue on the blood vessel wall to clean the lymph nodes, which prevented blood vessel from being damage. Finally, the gauzes strip can be blocked on the posterior wall of the stomach to block the stomach and omentum. The left hand maintained a certain tension, and the right-hand ultrasonic knife carried out naked cleaning along the blood vessels. The delta-shaped anastomosis was used both in Billroth - I (**Figure 3A**) and Billroth – II anastomosis (**Figure 3B**) in operation.

### 1.3 Postoperative management

During the postoperative period, patients were managed and cared on the basis of consensus guidelines for enhanced recovery after gastrectomy (11). Gastric or nutrition tubes were not used, and the urinary tube had been removed after awakening from anesthesia.

### 1.4 Observational indicators

Intraoperative and postoperative data, including operative time, intraoperative blood loss, postoperative pathological examination, number of lymph nodes dissected, postoperative hospitalization, first ambulation after surgery, time to first flatus, time to first water intake, abdominal drainage tube removal time, NRS pain score on the first postoperative day, hemameba level on the third postoperative day and postoperative complications, were recorded and analyzed.

## 2 Results

A total of 51 patients were included in the retrospective study. Among these patients, 21 patients (41.18%) were treated with TPLDG + D2 lymph node dissection, while the remaining 30 patients (58.82%) received FPLDG + D2 lymph node dissection. The clinicopathological characteristics of these patients are presented in **Table 1**. No intraoperative complications, conversion to laparotomy or added trocar were observed. We compared these two groups and found that there were no statistically significant differences in gender, age, TNM stage and BMI between the two groups, but the operation was strongly correlated with tumor size (*P* = 0.034) (**Table 1**).

**Table 1:** Patient demographics and baseline clinicopathological characteristics.

Characteristic	No of patients (N=51)	No of patients		*X*²	*P*-value
		TPLDG (21)	FPLDG (30)		
**Gender**				0.106	0.745
Male	40	16	24		
Female	11	5	6		
**Age (years)**				0.161	0.688
≥60	26	10	16		
<60	25	11	14		
**BMI (Kg/m^2^)**				2.596	0.107
≥21	31	10	21		
<21	20	11	9		
**Tumor size (cm)**				**5.789**	0.034*^a^
≥5	10	1	9		
<5	41	20	21		
**TNM stage**				0.496	0.635**^a^**
I-II	46	20	27		
III	5	1	3		

* Statistically significant difference; ^a^ Fisher's exact test.

In these two groups, 1 (0.05%) and 3 (10.00%) patients developed postoperative complications in the TPLDG and FPLDG groups, respectively (**Table 2**). There was one case of transient fever in the TPLDG group, in which the highest temperature was 38.2°C, but the patient’s condition was improved after physical cooling, which was considered as postoperative absorption of heat in this case. In the FPLDG group, patients who complained of discomfort and abdominal distension found relief after evacuation. Although the incidence of postoperative complications was lower in the TPLDG group than in the FPLDG group, no statistical difference was observed between the two groups (*P* = 0.635, **Table 2**).

**Table 2:** Comparison of postoperative complications between the TPLDG and FPLDG groups.

Characteristic	TPLDG group(21)	FPLDG group(30)	*P*-value
Postoperative complications	1 (0.05%)	3 (10.00%)	0.635^a^

a Fisher's exact test.

In addition, the operation time, intraoperative blood loss and the number of lymph nodes dissected should be counted in order to compare the intraoperative safety of the two groups (**Table 3**). However, there were no statistically significant differences between these two groups (*P* > 0.05) (**Table 3**).

**Table 3:** Comparison of intraoperative conditions between the TPLDG and FPLDG groups.

Characteristic	TPLDG group	FPLDG group	*t*-value	*P*-value
Operation time (mins)	224.80±9.44	232.20±5.97	0.697	0.489
Intraoperative bleeding (ml)	43.33±7.54	55.00±5.95	1.228	0.225
Number of lymph nodes examined	21.86±1.21	21.23±1.07	0.384	0.703

For the postoperative outcome, there were no obvious variations in the changes in the first ambulation, flatus, and water intake after surgery between the two groups (*P* > 0.05) (**Table 4**). However, the abdominal drainage tube removal time (1.62 ± 0.15 days vs. 2.00 ± 0.12 days, *P* = 0.046), NRS pain score on the first postoperative day (1.91 ± 0.15 days vs. 2.47 ± 0.12 days, *P* = 0.004) and hemameba level on the third postoperative day (7.89 ± 0.51 days vs. 10.52 ± 0.58 days, *P* = 0.002) were significantly lower in the TPLDG group compared to the FPLDG group (**Table 4**).

**Table 4:** Comparison of postoperative outcomes between the TPLDG and FPLDG groups.

Characteristic	TPLDG group	FPLDG group	*t*-value	*P*-value
Postoperative hospitalization (days)	4.14±0.28	4.37±0.17	0.726	0.471
First ambulation after surgery (hours)	11.71±0.67	13.07±0.63	1.445	0.155
Time to first flatus (days)	2.43±0.16	2.77±0.13	1.616	0.113
Time to first water intake (days)	1.10±0.07	1.20±0.07	1.003	0.321
Abdominal drainage tube removal time (days)	1.62±0.15	2.00±0.12	2.048	0.046*
NRS pain score on the first postoperative day	1.91±0.15	2.47±0.12	2.997	0.004*
Hemameba level on the third postoperative day (×10^9^)	7.89±0.51	10.52±0.58	3.208	0.002*

*Statistically significant difference.

## 3 Discussion

With the continuous improvement of laparoscopic surgery and the unremitting efforts of minimally invasive surgeons, the technology of laparoscopic reduction has gradually made a difference in modern medicine. It is famous for less disruption of the integrity of the abdominal wall, less postoperative pain, earlier ambulation, faster recovery, and fewer postoperative incision-related complications because only a small 3-4 cm incision was made around the umbilical cord for removal of the specimen, which improved cosmetic outcome and shorter hospital stay (2, 6). However, laparoscopic gastric cancer surgery *via* pore reduction revealed some disadvantages. Firstly, a dedicated multichannel port is required. Since the puncture hole is small, there will be crosstalk between forceps, while the lens and light sources may interfere with the operator’s left-hand forceps or the assistant’s forceps, affecting the field of view, leading to a slower operation, or even abandoning the hole reduction procedure. Secondly, there are certain difficulties in conventional laparoscopic techniques to reduce laparoscopy by following the “triangular operation principle”, especially in uniportal conditions, where the “ chopstick effect “ occurs between individual parallel instruments, making surgical manipulation difficult (9, 12). Finally, due to the lack of assistance, the intraoperative process can only be advanced by the coordination of both the main surgeon and the assistant, which caused the difficulty of the reduced hole laparoscopic technique and longer operations time. This difficulty is particularly obvious in the single-hole laparoscopic technique.

On the basis of the conventional five-hole method but the reduction of the two operating holes of the assistants, there is no use of a multichannel puncher and no change in the placement of other instruments. We hereby provide crucial tips on performing TPLDG. Firstly, for omental resection operation, the left-hand duckbill forceps lifts the omentum ventrally and cephalic, using the traction of the left-hand duckbill forceps and the gravity of the natural descent of the transverse colon to maintain a moderate tension to provide the anatomical space. Then, the avascular area along the attachment of the omentum at the edge of the transverse colon extends from the middle to the left and right sides, which prevents the colon wall from thermal injury by the ultrasound knife. The whole procedure should be performed carefully because there may be a case of indefinite adhesions at the time of dissociation of the splenic and hepatic flexures of the colon. The omentum can be pulled by a left-hand grasping forceps to form different angles in different directions, and both blunt and sharp separations using an ultrasonic knife can be performed. The colon can be pressed under the nonworking surface of the ultrasonic knife to better discern the gap, which avoids clamping the tissue into large pieces and disconnects again after identifying the surrounding tissues that the ultrasonic knife head may touch. Secondly, for lymph node dissection, since the root of the right gastroepiploic vessel lies slightly right of the midline, a left-sided approach may be used for lymph node dissection in group 6. It is safe and feasible for us to perform TPLDG+D2 lymph node dissection in our clinical practice. For D2 lymph node dissection, the splenic vein, artery, and portal vein must be revealed, and 11p and 12a lymph node dissections should be performed, respectively. After the dissection of lymph nodes in group 6, the duodenum was cut, the right gastric vessel was cut and ligated at the root, and the lymph nodes in group 5 were cleared (**Figure 2B**). Using the vessel as a guide, the left duckbill forceps pulled the lymphatic tissue of the anterior wall of the common hepatic artery, and a scalpel was used to press the hepatic artery in the direction of the portal vein (**Figure 2C**). The ultrasound knife was used when separation forceps were used, with careful blunt separation to reveal the portal vein. Both 8a and 12a lymph nodes were dissected completely. Then, a left dissection was conducted to complete lymph node dissection in groups 7 and 9. The posterior mesentery of the stomach was pulled with the left hand, which continued a sharp cephalic dissection along the pre-renal fascia to both diaphragmatic feet. The left-hand grasping forceps lifted the posterior wall of the stomach and the cephalic dissection continued along the left quaternary rib region. Regarding the removal of group 11p lymph nodes, the procedures were begun with dissociation along the proximal splenic artery and exposure to proximal splenic artery and vein (**Figure 2D**). In this action, it is important not to damage the posterior gastric artery and vein.

We believe that the three-hole operation method can completely isolate the main knife operation hole, and avoid the interaction with the hand-holding mirror. The left side station is more consistent with the five-hole method station which led to the quick adaptation to the operation rhythm between the operator and assistant. Furthermore, no other additional special instruments and costs are required. If encountering some urgent difficulties with the three-hole operation, it is possible to increase the number of instruments at any time and convert to conventional laparoscopic surgery. In this way, the problems of instrument interference, “coaxial effect”, reduced field of view and distress of the triangular plane could be greatly handled. Moreover, this technique is more conducive to fine manipulation. The three-hole method operation requires that the operator has a more demanding control at the surgical level for gastric cancer resection, and there is no assistant side injury, which does not affect the advantage of minimally invasive surgery. Our study found that the TPLDG is safe and feasible, and even has advantages over the FPLDG in terms of postoperative recovery, such as drainage removal time, postoperative pain score, and inflammatory indicators.

In conclusion, TPLDG was developed based on traditional reduced port laparoscopic surgery. The clinical procedure is feasible and safe, and the results herein presented are satisfactory. However, the application of new technology must be performed on the premise of ensuring the safety of the patient, therefore we will continue to expand the number of surgeries performed, and gradually carry out prospective, multicenter, large-sample, randomized controlled clinical trials, in order to facilitate the promotion of this technique and improve the standardization and operability to benefit more eligible patients with gastric cancer.

## Data availability statement

The raw data supporting the conclusions of this article will be made available by the authors, without undue reservation.

## Ethics statement

Written informed consent was obtained from the individual(s) for the publication of any identifiable images or data included in this article.

## Author contributions

GJ and JW designed the experiments and wrote the paper. XY and XL performed statistical analyses. RG and WW provided expression data and patient sample material. All authors contributed to the article and approved the submitted version.
